# The Field of Science

**Published:** 1894-03-24

**Authors:** 


### THE FIELD OF SCIENCE.

Social democracy is not encouraged among the medical faculty of Leipsig; in fact, only recently the local Medical Society have declared that no member will be eligible for this body who is in avowed confraternity with Social Democrats.

The Church Sanitary Association is recommending that the Seventh Sunday after Trinity shall be a "Sanitation Sunday," on which the hygienic necessity of personal cleanliness, fresh air, abundant light, pure water and food, and safeguards against infectious diseases shall be universally spoken of from the British pulpit.

A crematorium for the disposal of the bodies of victims to cholera has been erected at Cardiff. This is the work—and an excellent one, too—of the local Port Sanitary Authority. For the comparatively small fee of £7 for each body, the advantages of the crematorium are also offered to the Bristol and Gloucester port districts. Infected excreta, clothing, and waste refuse from the cholera hospital will also here have facilities of destroyal.

The Revue d'Hygiène is urging the necessity of further measures being adopted against the contagion of phthisis. For instance, it is suggested that the French hospitals shall in future adopt the precautions noticeable in our English ones regarding the sputa of phthisical patients. Baron Larrey is pressing on the Paris Academy of Medicine to create a prize for tuberculosis statistics, with the view of encouraging further investigation on the mortality from phthisis.

It says something for the work the Pasteur Institute has done at Constantinople when we learn on the authority of a Turkish paper that the sphere of its usefulness is to be further extended throughout the country. An Imperial "iradé" has just been issued commanding that in the chief town of each province a similar antirabific laboratory shall be established, similar in plan to the one now existing in the Turkish capital. These fresh branches of the Pasteur Institutes will now be established in the most distant provinces of the Sultan's domains, amongst which towns will be Jemen, Bagdad, and Damascus.

The Bill for the establishment of a National Bureau of Health in America has been laid before both Houses of Congress. We gather that the chief medical societies of the country, besides the President and the Secretary of the Treasury, are in favour of the scheme. Universal sympathy has been expressed with the movement, and it is to be hoped that the long-deferred organization of a National Bureau of Health will soon assume a definitely tangible form.

A chorus of condemnation has hailed the proposed foundation at Chelsea of an Institute for scientific research. The inhabitants are full of the deadly evils menacing its establishment, amongst which dangers a correspondent urges that flies will carry off deadly bacilli from the neighbouring laboratory and scatter them broadcast around. This Institute has further been called the "New Torture Chamber"; and, as a contemporary wisely comments: "Of course, to be consistently logical, all good sentimentalists who are so eager to denounce the inhuman brutality of vivisectionists must decline to avail themselves of any advantages that have resulted from that practice!"

"Flesh taken from a man's arm, to make ears for another," is the thrilling title of a story which reaches us from Belgium, and, sensational as the anecdote is, yet it has something authentic about it. It appears that quite lately a Belgian was confined in a prison cell with another inmate whose condition bordered on delirium tremens. This amiable companion in captivity turned upon the intruder, attacking him furiously and eventually biting off the fellow-prisoner's ears. The furious combat was terminated on the entrance of the gaoler, and the victim's quarters were speedily changed for that of a hospital ward. Later on the patient was visited by his former assailant, who appeared now under the guise of the penitent drunkard. Dr. Laudry, who was standing near the bed, asked the latter, "Should you like to give him back his ears?" "Certainly," replied the penitent. "Then," said the doctor, "let me cut out a couple of small slips of flesh from your arm and the thing is done." To this the man readily assented, whereupon Dr. Laudry, report goes, performed a plastic operation with complete success.

